# Supplementary material for: Chemically induced senescence in human stem cell‐derived neurons promotes phenotypic presentation of neurodegeneration
Source: Aging Cell. 2021 Dec 24;21(1):e13541. doi: 10.1111/acel.13541 (PMC8761019; doi:10.1111/acel.13541)
Supplement: Supplementary file 1 — Supinfo S1 [file ACEL-21-e13541-s001.pdf]

Supplementary Information

Chemically Induced Senescence in Human Stem Cell-Derived Neurons Promotes Phenotypic Presentation of Neurodegeneration

Ali Fathi[1,6], Sakthikumar Mathivanan[1,6], Linghai Kong[1], Andrew J Petersen[1], Cole R. K. Harder[1], Jasper Block[1], Julia Marie Miller[1], Anita Bhattacharyya[1,2], Daifeng Wang[1], Su-Chun Zhang[1,3,4,5*]

1- Waisman Center, School of Medicine and Public Health, University of Wisconsin-Madison, Madison WI 53705, USA
2- Department of Cell and Regenerative Biology, School of Medicine and Public Health, University of Wisconsin-Madison, Madison WI 53705, USA
3- Department of Neuroscience, School of Medicine and Public Health, University of Wisconsin, Madison, WI 53705, USA
4- Department of Neurology, School of Medicine and Public Health, University of Wisconsin, Madison, WI 53705, USA
5- Program in Neuroscience & Behavioral Disorders, Duke-NUS Medical School, Singapore, Singapore
6- These authors contribute equally.

*Correspondence: suchun.zhang@wisc.edu

Figure S1

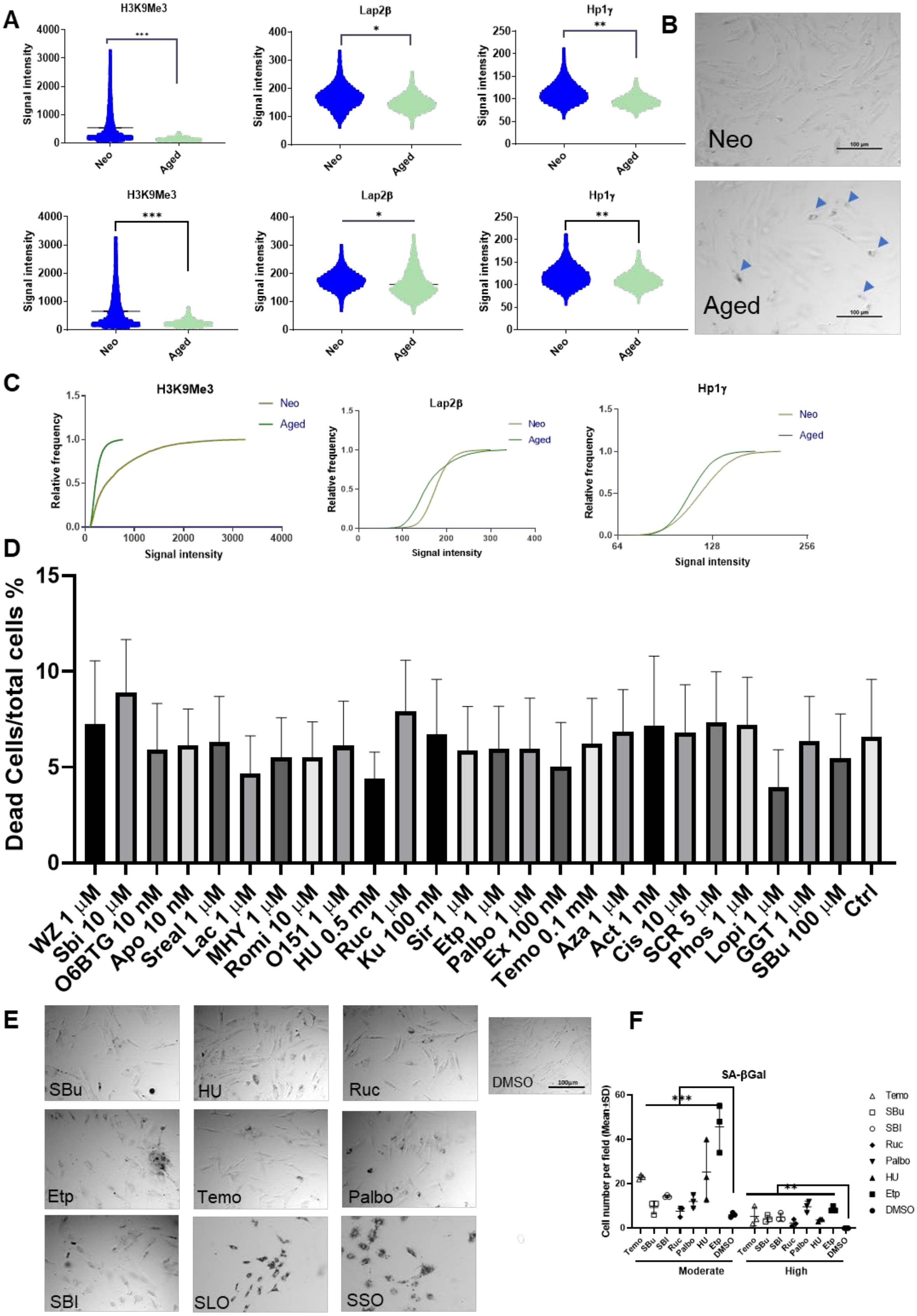

A
H3K9Me3
Lap2β
Hp1γ
Signal intensity
Neo
Aged
B
Neo
Aged
C
Relative frequency
Signal intensity
D
Dead Cells/total cells %
WZ 1 μM
Sbi 10 μM
O6BTG 10 nM
Apo 10 nM
Sreal 1 μM
Lac 1 μM
MHY 1 μM
Romi 10 μM
O151 1 μM
HU 0.5 mM
Ruc 1 μM
Ku 100 nM
Sir 1 μM
Etp 1 μM
Palbo 1 μM
Ex 100 nM
Temo 0.1 mM
Aza 1 μM
Act 1 nM
Cis 10 μM
SCR 5 μM
Phos 1 μM
Lopi 1 μM
GGT 1 μM
SBu 100 μM
Ctrl
E
SBu
HU
Ruc
DMSO
Etp
Temo
Palbo
SBI
SLO
SSO
F
SA-βGal
Cell number per field (Mean±SD)
Temo
SBu
SBI
Ruc
Palbo
HU
Etp
DMSO
Moderate
High

Figure S1. Related to figure 1, (A) Individual values for H3K9Me3, Lap2β and HP1γ expression in both male (upper panel) and female (lower panel) fibroblast cells and (B) phase contrast images of senescence associated β-Galactosidase staining (arrowheads) for both neonatal and aged (female 62 years old) fibroblasts. (C) Frequency distribution from high content imaging for H3K9Me3, Lap2β and HP1γ proteins in female neonatal and aged (62 years old) fibroblasts. (D) Cell toxicity assay for small molecules in the used concentration in actual experiments compared to the DMSO control in neonatal fibroblasts. (E) Phase contrast images of senescence associated β-Galactosidase staining for top seven molecules that induced senescence and SLO and SSO combinations in neonatal fibroblasts, and (F) quantification results for percentage of positive cells (all numbers across replicates pooled) and divided to high expression and moderate expression classes based on intensity of staining. (n=3, *: $p<0.05$, **: $p<0.01$, ***: $p<0.001$ one-way ANOVA with Dunnett's multiple comparison test). Scale bar = 100um.

Figure S2

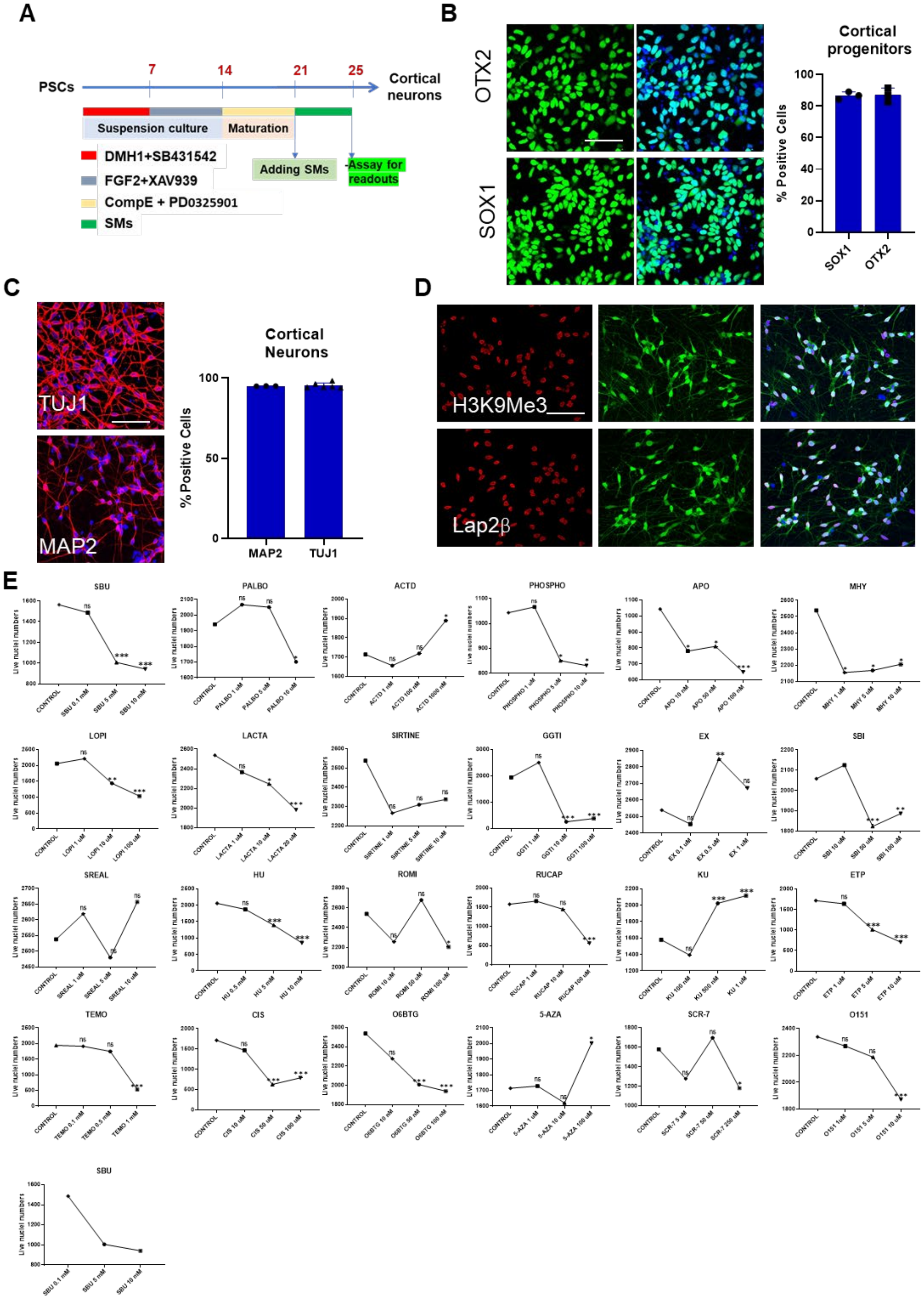

Figure S2. related to figure 3- differentiation of cortical neurons from H9-GFP ESCs and characterization for expression of neuronal markers.

(A) Differentiation protocol used for generating cortical neurons from H9-GFP stem cells. (B) Immunostaining images for SOX1 and OTX2 in day-14 cortical progenitors and quantification for proportion of positive cells. Scale bar=50 μm. (C) Representative immunostaining images for day-21 cortical neurons expressing TUJ1 (TUBB3) and MAP2 proteins in red and nuclei stained with Hoechst in blue (Scale bar=100 μm) and quantification for percentage of positive neurons. (D) Immunostaining images for H3K9Me3 and Lap2β proteins in day-21 GFP labeled cortical neurons, Scale bar=100 μm. (E) Cell toxicity assay for different doses of 25 small molecules in cortical neurons. (n=3, ns: not significant, *: p<0.05, **: p<0.01, ***: p<0.001 one-way ANOVA with Dunnett's multiple comparison test).

Figure S3

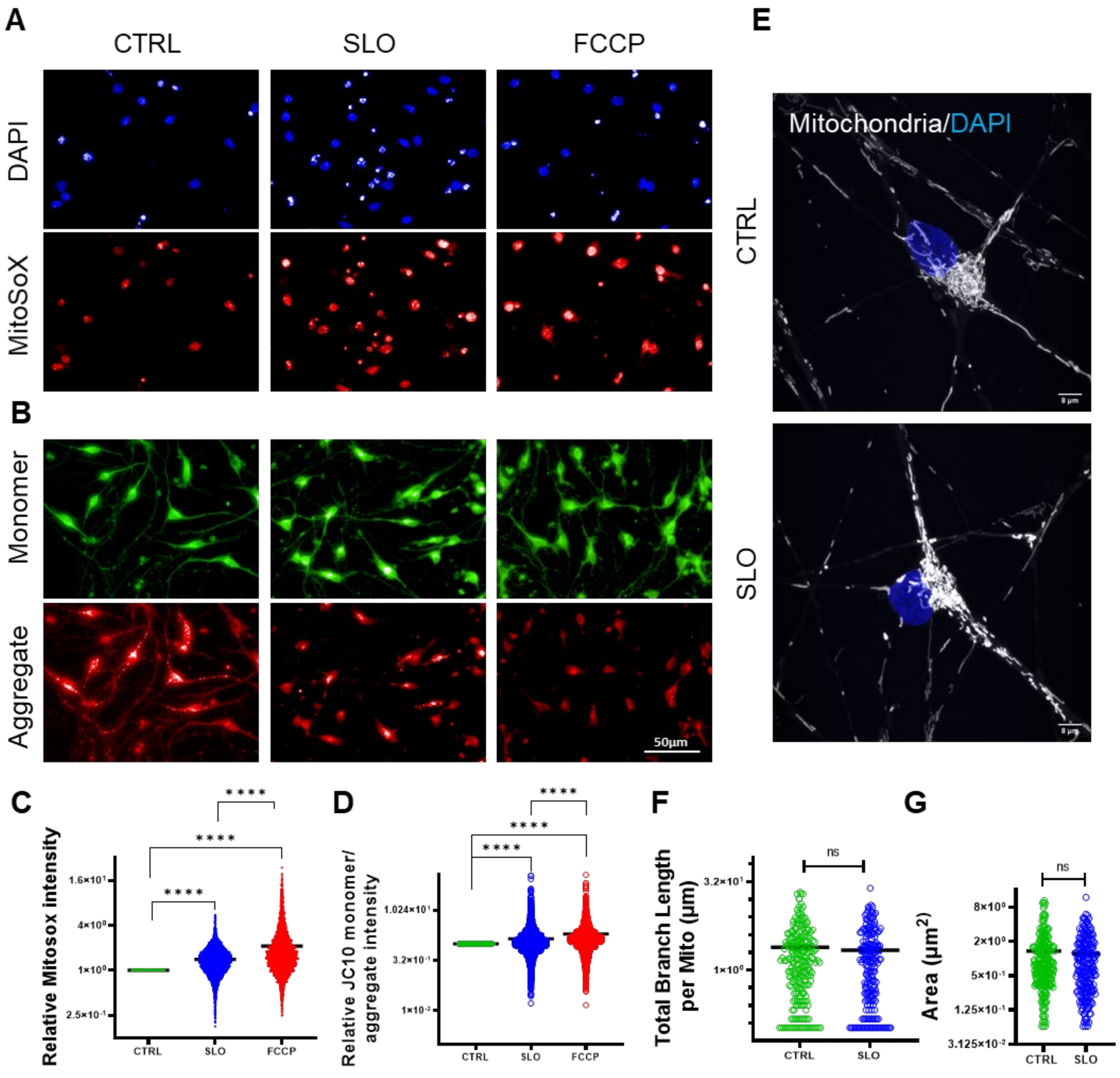

Figure S3. MitoSoX and Mitochondrial membrane potential in SLO treated cortical neurons. Representative images of cortical neurons at day 25 for (A) MitoSoX staining and (B) JC-10 fluorescence (Top- Monomer (green), Bottom- Aggregate (red) from Control, SLO and FCCP treated cortical neurons). Scale bar= 50μm. (C) Statistical analysis of relative MitoSoX intensity from Control, SLO and FCCP treated neurons. (D) Statistical analysis of relative JC10 Monomer to Aggregate intensity from Control, SLO and FCCP treated neurons (Low monomer to aggregate ratio means high mitochondrial membrane potential, high monomer to aggregate ratio means low mitochondrial membrane potential). Represented images of (E) Mitotracker stained mitochondria in cortical neurons and quantification results for (F) mitochondrial length and (G) mitochondrial area in SLO treated neurons versus control neurons. Data were collected using 15,000 cells per group from three independent experiments. Statistical analysis was performed using One-way ANOVA, Tukey post-hoc test (n=3, ****- P<0.001).

Figure S4

A

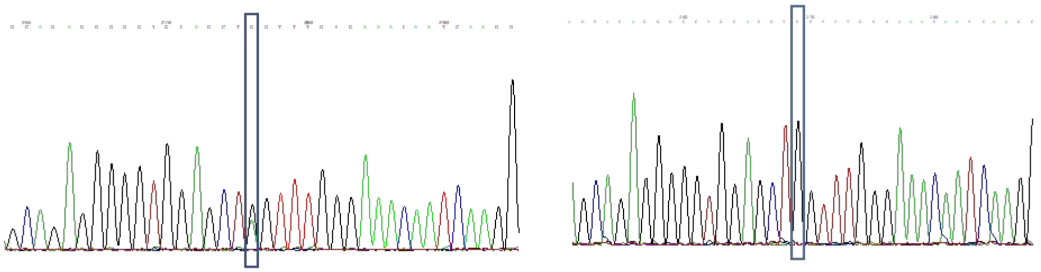

B

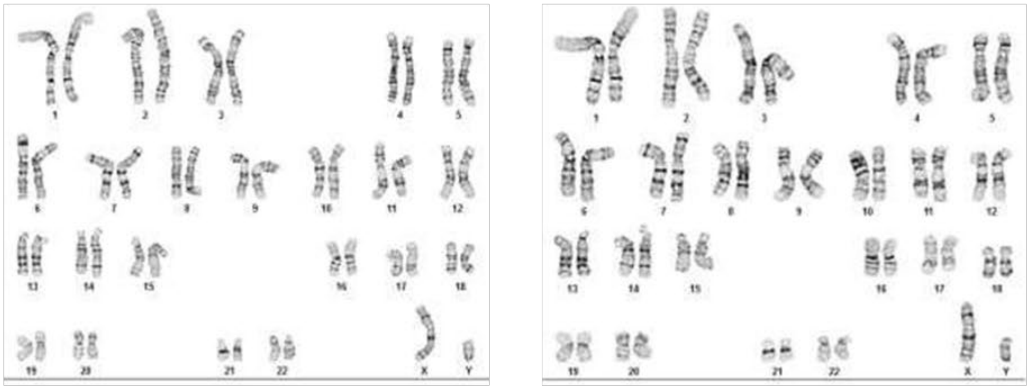

TDP43 G298S iPSC#13	*TDP43 G298G iPSC#19*
Total Counted: ***20***	***Total Counted:*** ***20***
Total Analyzed: ***8***	***Total Analyzed:*** ***8***
Total Karyogrammed: ***4***	***Total Karyogrammed:*** ***4***
Band Resolution: ***400 - 500***	***Band Resolution:*** ***475 - 550***

C

STR Locus	STR Genotype Repeat #	STR Genotype
FGA	16–18,18.2,19,19.2,20,20.2,21,21.2,22, 22.2, 23, 23.2, 24, 24.2, 25, 25.2, 26–30, 31.2, 43.2, 44.2,45.2, 46.2	19,21
TPOX	6-13	9,11
D8S1179	7-18	10,12
vWA	10-22	17,17
Amelogenin	X,Y	X,Y
Penta_D	2.2, 3.2, 5, 7-17	11,12
CSF1PO	6-15	10,13
D16S539	5, 8-15	9,11
D7S820	6-14	8,12
D13S317	7-15	11,12
D5S818	7-16	11,14
Penta_E	5-24	7,16
D18S51	8-10, 10.2, 11-13, 13.2, 14-27	12,16
D21S11	24,24.2,25,25.2,26-28,28.2,29,29.2, 30, 30.2,31, 31.2,32,32.2,33,33.2, 34,34.2,35,35.2,36 -38	30,31
TH01	4-9,9.3,10-11,13.3	6,9.3
D3S1358	12-20	15,15

STR Locus	STR Genotype Repeat #	STR Genotype
FGA	16–18,18.2,19,19.2,20,20.2,21,21.2,22, 22.2, 23, 23.2, 24, 24.2, 25, 25.2, 26–30, 31.2, 43.2, 44.2,45.2, 46.2	19,21
TPOX	6-13	9,11
D8S1179	7-18	10,12
vWA	10-22	17,17
Amelogenin	X,Y	X,Y
Penta_D	2.2, 3.2, 5, 7-17	11,12
CSF1PO	6-15	10,13
D16S539	5, 8-15	9,11
D7S820	6-14	8,12
D13S317	7-15	11,12
D5S818	7-16	11,14
Penta_E	5-24	7,16
D18S51	8-10, 10.2, 11-13, 13.2, 14-27	12,16
D21S11	24,24.2,25,25.2,26-28,28.2,29,29.2, 30, 30.2,31, 31.2,32,32.2,33,33.2, 34,34.2,35,35.2,36 -38	30,31
TH01	4-9,9.3,10-11,13.3	6,9.3
D3S1358	12-20	15,15

D

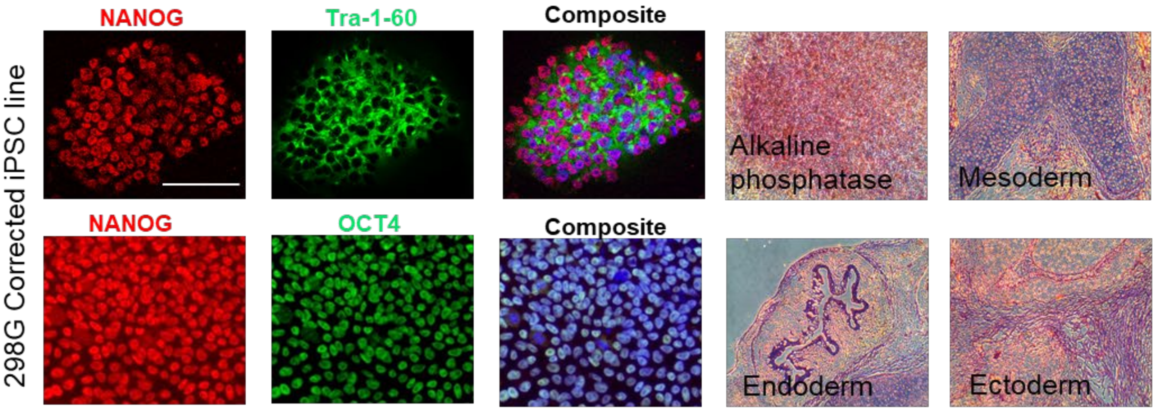

Figure S4. Related to figure 6- Characterization of TDP43 G298S and TDP43 G298G iPSCs.

(A) Sanger sequencing result for mutant (left panel) and corrected (right panel) (isogenic control) cell lines. (B) Karyotype analysis for mutant (left) and corrected (right) cell lines. (C) STR analysis for both cell lines were done for selected loci. (D) Immunostaining for Nanog, Tra1-60, and Oct4 as well as Alkaline phosphatase staining and HE staining, showing endoderm, ectoderm and mesoderm like structures in TD43 G298S corrected line.

Figure S5

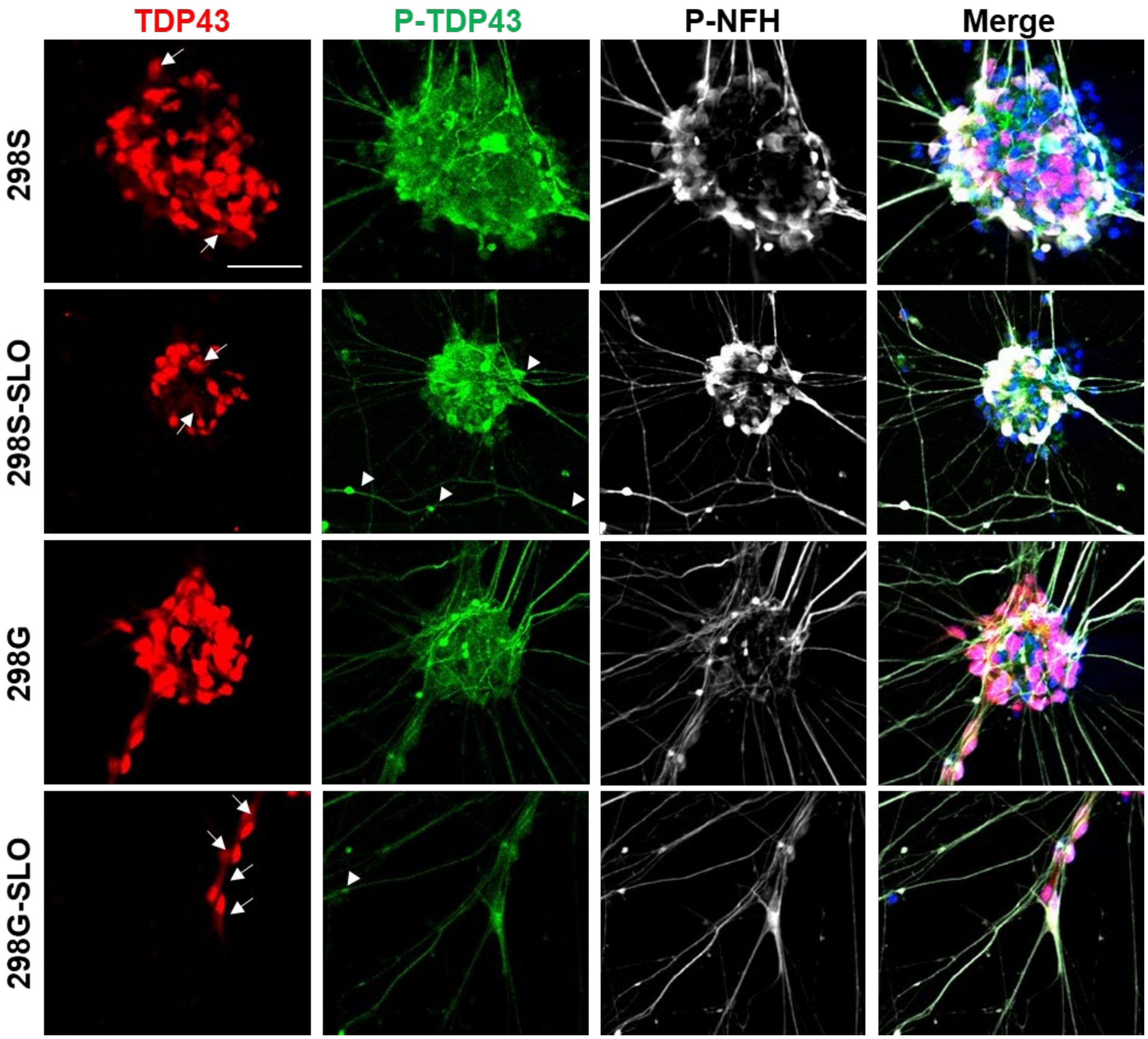

Figure S5. Related to Figure 6- ALS MNs treated with SLO molecules shows signs of neurodegeneration and protein phosphorylation. Immunostaining images of phosphor-TDP43 protein following SLO treatment in 298S mutant cells and 298G healthy control cells, arrow heads

are pointed at p-TDP43 positive neurite swellings and positive signals in the nuclei region of MNs, arrows are pointed at positive signal for TDP43 in the cytoplasmic region of MNs (Scale bar=100 μm),

Figure S6

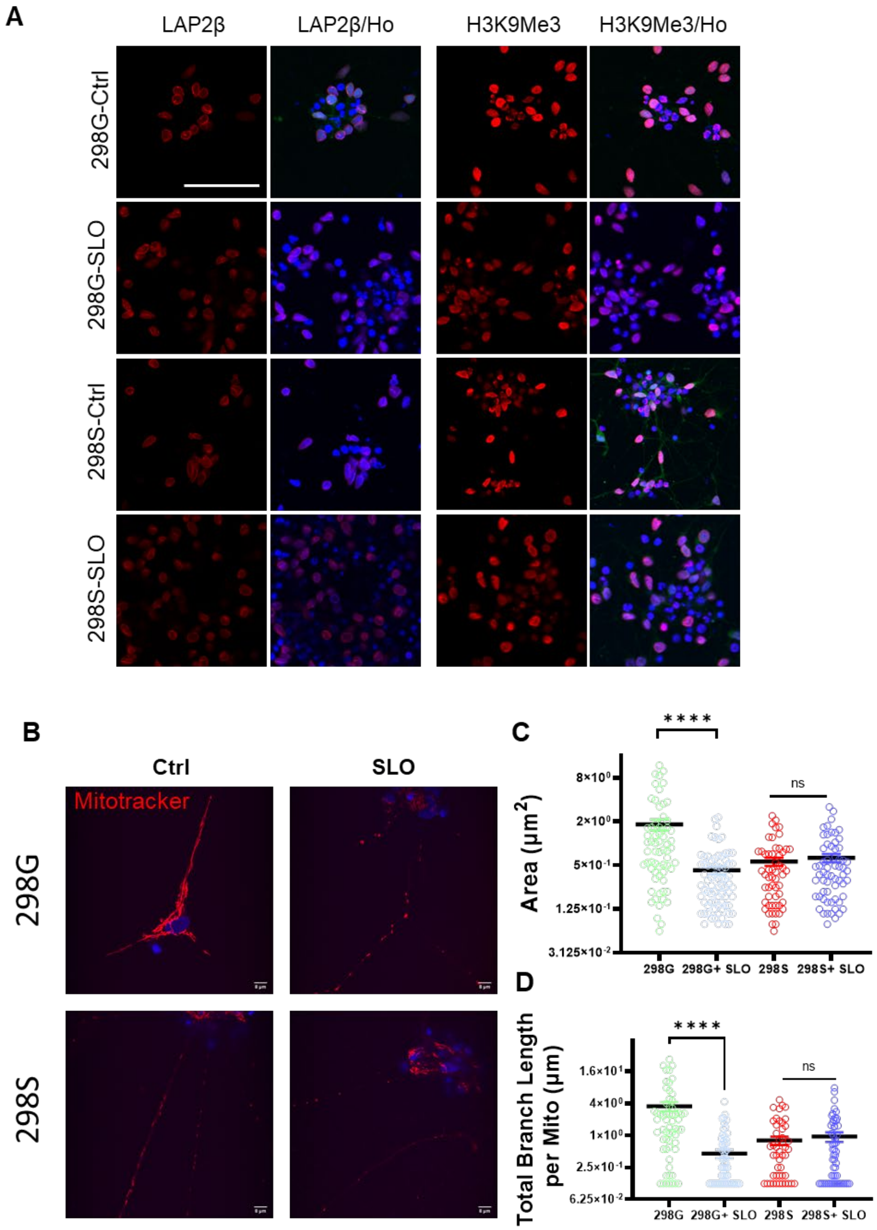

Figure S6. Related to Figure 6. (A) Immunostaining images of LAP2β and H3K9Me3 following SLO treatment in 298S mutant cells and 298G healthy control cells. (B) MNs stained for Mitotracker red to visualize morphological changes in SLO treated cells (Scale bar=8 μm), and

graphs of quantified data for (C) mitochondrial area and (D) total branch length for each mitochondria. (n=3*: p<0.05, ***: p<0.001 one-way ANOVA with Dunnett's multiple comparison test).

Figure S7

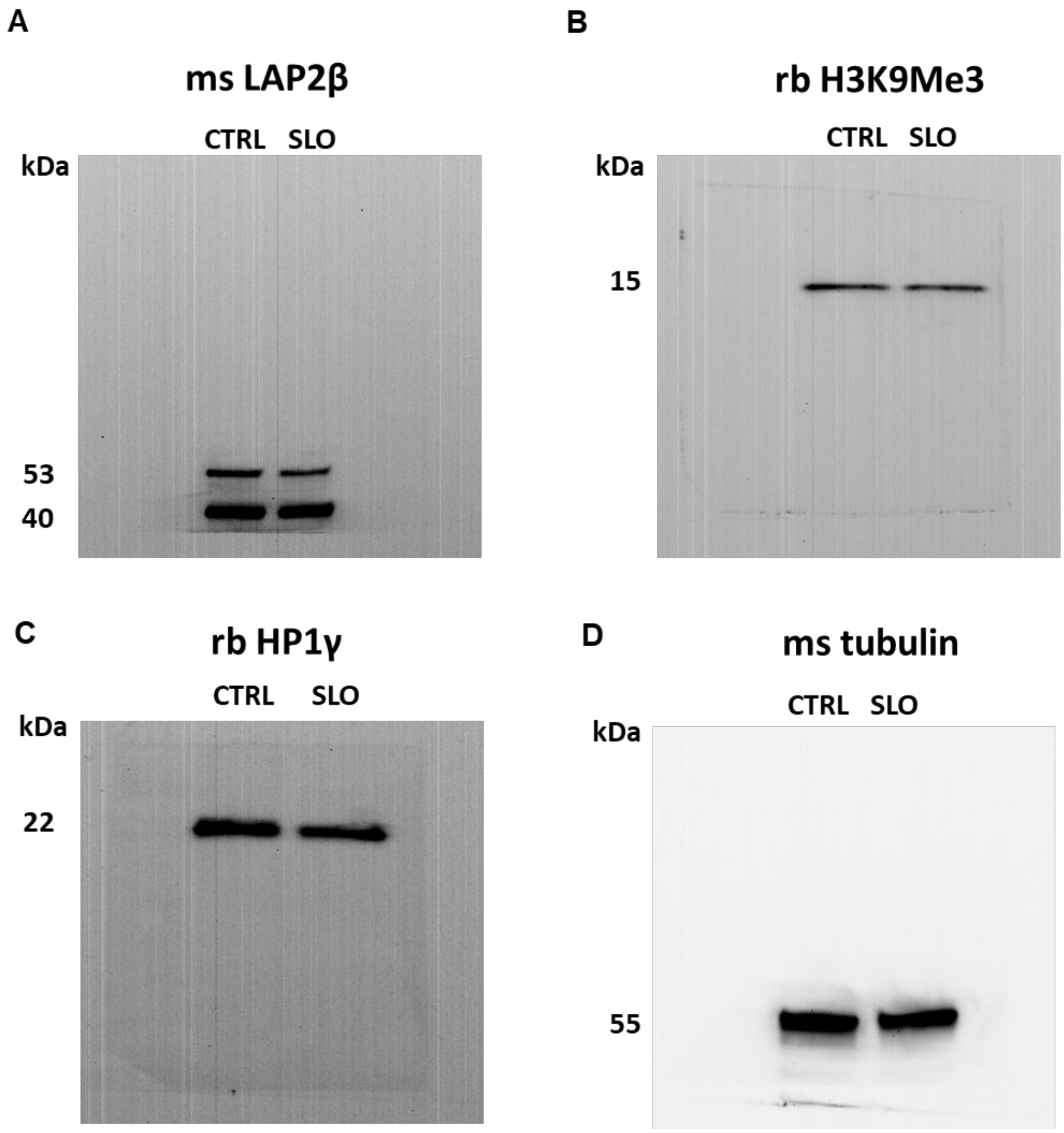

Figure S7. Related to Figure 4G. Representative images of uncropped western blot for (A) LAP2β, (B) H3K9Me3, (C) HP1γ and (D) Tubulin in cortical neurons treated at day 21 of differentiation

Table S1: List of the small molecules and their concentration for final screen in neurons.					
**Name**	Function	Working Concentration	Name	Function	Working Concentration
**WZ4003**	AMPK inhibitor	2μM	**SirReal2**	Sirt2 inhibitor	1μM
**MHY1485**	mTOR activator	2μM	**Rucaparib**	PARP1 inhibitor	1μM
**Fumonisin B1**	AKT activators	5μM	**Temozolomide**	DNA alkylation	100μM
**Sirtinol**	Sirtuin inhibitors	5μM	**Lactacystin**	irreversible proteasome inhibitor	4nM
**SBI-0206965**	Autophagy inhibitor	10μM	**KU-60019**	ATM inhibitor	100nM
**Romidepsin**	HDAC1,2 inhibitor	10pM	**5-AZA-20-DEOXYCYTIDINE**	DNA methyltransferase inhibitor	1μM
**Etoposid**	Topo II inhibitor	2μM	**Actinomycin D**	inhibiting DNA-primed RNA synthesis	10nM
**Lomeguatrib-O6BTG**	MGMT inhibitor	10nM	**Cisplatin**	Topo I,II inhibitor	10μM
**O151**	DNA Glycosylase-1 inhibitor	1μM	**SCR-7**	Ligase V inhibitor	5μM
**Palbociclib**	CDK4/6 inhibitor	1μM	**Phosphoramidon**	metalloendopeptidase inhibitor	1μM
**Apo866**	NAD biosynthesis inhibitor	10nM	**Lopinavir**	HIV protease inhibitor	1μM
**Hydroxyurea**	DNA synthesis stress inducer	500μM	**GGTI298**	geranylgeranyltransferase I (GGTase I) inhibitor.	1μM
**EX-527**	Sirt1 inhibitor	100nM	**Sodium Butyrate**	histone deacetylase inhibitor I, II	100μM
**SMER28**	Autophagy activator	1μM	**Edaravone**	Radical scavenger	1μM
**Tat-Beclin**	Autophagy activator	100nM	**Amiodarone**	K^+ channel blocker	5μM
**STF-62247**	Autophagy activator	1μM	**Flubendazole**	Autophagy activator	1μM
